# Famotidine promotes inflammation by triggering cell pyroptosis in gastric cancer cells

**DOI:** 10.1186/s40360-021-00533-7

**Published:** 2021-10-22

**Authors:** Jin Huang, Pingsheng Fan, Miao Liu, Chengtao Weng, Gaofei Fan, Tengyue Zhang, Xiaohong Duan, Yang Wu, Lili Tang, Guohong Yang, Yabei Liu

**Affiliations:** Department of Medical Oncology, the First affiliated Hospital of University of Science and Technology of China(West), Heifei, Anhui China

**Keywords:** Famotidine, Cell pyroptosis, GSDME, gastric cancer cells

## Abstract

**Background:**

Cell pyroptosis has been characterized by cell swelling and pro-inflammatory factors release to aggravate inflammatory reaction., such as interlukin-1 beta (IL-1β) and interlukin18 (IL-18). However, the function of famotidine, an antagonist of histamine H2-receptor antagonists, in cell pyroptosis remained unknown.

**Methods:**

Real-time quantitative PCR (qPCR), western blotting (WB), LDH release assay and enzyme linked immunosorbent assay (Elisa) combined with inhibitor were performed to analyze the effect of famotidine on cell pyroptosis-related gene expression.

**Results:**

In this study, we found that famotidine (300 μm) treatment led to a phenomenon of cell pyroptosis as confirmed by LDH assay. Further results showed that famotidine triggered cell pyroptosis in gastric cancer cells by activation of NLPR3 inflammasomes including ASC, Caspase-1 and NLRP, leading to enhanced IL-18, not IL-1β, mature and secretion. What’s more, the results also showed GSDME, not GSDMD, was increased in response to famotidine stimulation in BGC823 and AGS cells. Mechanically, phosphorylation of ERK1/2 was drastically enhanced in present with famotidine treatment, while inhibition of ERK1/2 activity by U0126 could reverse the promotion of famotidine in IL-18 secretion.

**Conclusion:**

These findings revealed a novel role of famotidine in cell pyroptosis in patients with gastric cancer, a comprehensive consideration is needed in treatment of gastric cancer.

## Background

Gastric cancer, a malignancy with increasing incidence and mortality, has been the leading cause of death overall the world [1–3], which has been listed as a worldwide principal health issue[4–6]. At present, surgery remains the only curative treatment for gastric cancer, while chemotherapy is considered as an effective supplementary therapy to prevent recurrence and metastasis following surgical excision[1, 7]. According to histotype, gastric cancer included adenocarcinoma, anaplastic carcinoma, mucinous carcinoma, specific type of cancer such as adenosquamous cell carcinoma, squamous cell carcinoma. The human gastric cancer cells, including BGC823 (gastric carcinoma cell line) and AGS (gastric adenocarcinoma cell line), were used to explore the gastric cancer progression in vitro[8, 9]. Recently, the studies have demonstrated gasdermin (GSDM)-mediated pyroptosis have a critical role in the mechanism of pathogenesis and chemotherapy underlying GC, including proliferation, immune responses, even in chemotherapy resistance[10–15], highlighting that targeting gasdermin family expression is an improvement of therapeutic approaches for gastric malignant disorders. However, the regulation of cell pyroptosis remains to be fully elucidated.

Cell pyroptosis, also called inflammatory programmed cell death and characterized by NLRP inflammasomes, may lead to host cells death depending on the microenvironment stimulation[16, 17], which is featured by inflammatory caspases activation, including caspase-1/-11/-4/-5[18], leading to convert the pro-forms of interleukin (IL)-18 and IL-1β into mature inflammatory[18–22]. In general, the NOD-like receptors (NLPs), especially NLR family pyrin domain containing 3 (NLRP3), recognize intracellular pathogens and form NLRP3 inflammasome activation via association with the adaptor protein ASC, leading to caspase-1 activation, which further cleaves pro-IL-1β and pro-IL-18 into inflammatory IL-1β and IL-18 forms. In addition, activated caspase-1 also cleaves gasdermin D (GSDMD) that enables cell membrane pore-formation for the release of IL-1β and IL-18 and triggering of pyroptosis[23]. In addition to pyroptosis, either intrinsically or extrinsical stimulation led to apoptosis, which was attributed to the executioner caspases 3 and 7 (CASP3, CASP7) activation, resulting in proteolysis, nuclear fragmentation, and apoptotic cell death[24], while necroptotic cell death was initiated by death receptors (DRs), pattern recognition receptors (PRRs), and the necrosome formation was consisted of receptor-interacting protein kinase-1 (RIPK1) and RIPK3, which was kept in check by CASP8-mediated cleavage of RIPK1 and RIPK3, such that in the presence of CASP8, the cell death mode defaults to apoptosis[25, 26]. Auto-phosphorylation of RIPK3 recruited and activated MLKL to the necrosome (pMLKL). Which further translocated to the plasma membrane where it forms a tetramer and permeabilizes the cell membrane causing oncolysis. Interestingly, abnormal expression of NLRP3 and GSDMD is increased in gastric cancer tissue promotes GC cells proliferation and tumorigenesis via inducing Cyclin D1 (CCND1) and cell cycle-related proteins expression[13, 27], while high expression of GSDME in gastric cancer cells undergo the switch from caspase-3 dependent apoptosis to pyroptosis induced by chemotherapeutic drugs[15]. These findings demonstrated that the pyroptosis is critical in development of gastric cancer.

Gastric antisecretory drugs, including proton pump inhibitors (PPIs) and histamine H2 receptor antagonists, are widely used among patients with cancer in clinical practice. Pervious study have demonstrated that omeprazole suppressed *de novo* lipogenesis in gastric epithelial cells[28], and rabeprazole inhibited cell proliferation in gastric epithelial cells by targeting STAT3-mediated glycolysis[29]. While famotidine, a histamine H[2] receptor antagonist, has been reported to protect against cell death induced by MK-801 through glycogen synthase kinase 3 β (GSK-3β)/β-catenin signaling pathway in SK-SY5Y cells[30]. Further study showed that the novelty and importance of the famotidine in the management of cardiovascular diseases through NF-κB[31]. What’s more, famotidine treatment in the sinusoidal capillaries increased cell infiltration, promoted fatty degeneration of hepatocytes, and exacerbated dilatation[32]. However, there are no adequate reports or only rare reports of its use in patients with gastric cancer. Herein, we further showed that famotidine stimulation in gastric cancer cells of BGC823 and AGS significantly promoted cell pyroptosis by inducing NLRP3 activation, leading to caspase-1 activation, IL-1β and IL-18 release. This phenomenon is attributed to increased phosphorylation of ERK1/2 caused by famotidine. These finding extended the novel function of famotidine in gastric cancer cells, which would aid the selection of sedation for patient’s treatment.

## Methods

### Cell culture, treatment, and reagents

The human gastric cancer cells, including BGC823 (gastric carcinoma cell with poor differentiation) and AGS (gastric adenocarcinoma cell with well differentiation), were purchased from the American Type Culture Collection (ATCC, Manassas, VA, USA) and cultured in Eagle’s minimal essential medium (EMEM) supplemented with 10 % fetal bovine serum (FBS, Gibco, USA), which have employed as *in vitro* cell model to study cell pyroptosis [12, 33]. All the cells were cultured under standard culture conditions with 37 ℃ humidified air containing 5 % CO_2_. As for treatment, cells were stimulated with or without 300 μm famotidine in study. Medium and fetal bovine serum were purchased from life technologies. Pierce™ BCA protein assay Kit and PageRuler™ Prestained Protein Ladder were from thermo fisher, trizol was from invitrogen (Invitrogen, Thermo Fisher Scientific). All-in-one™ first-strand cDNA synthesis kit and All-in-one™ qPCR mix were from Genecopoeia™, FulenGe. Famotidine (S2078) and caspase-1 inhibitor Belnacasan (VX-765) (S2228) was purchased from Selleck. Other chemical reagents were from Sigma. The antibodies NLRP3(Abcam; ab260017, 1:2000 for WB), IL-18(Abcam; ab235697, 1:1000 for WB), IL-1β (Abcam; ab216995, 1:1000 for WB), ASC (Abcam; ab151700, 1:2000 for WB), GSDMD (Abcam; ab210070, 1:2000 for WB), GSDME (ab215191, 1:1000 for WB), p-ERK1/2(Abcam; ab242418, 1:1000 for WB) and ERK1/2(ab148699, 1:1000 for WB) were purchased from Abcam. Human IL-18 ELISA Kit (ab215539) and Human IL-1 β ELISA Kit (ab217608) were purchased from Abcam. VX-765(S2228) was purchased from Selleck Chemicals.

## Quantitative real-time PCR

As described in Zhang et al study[34], the total RNA was extracted to converted from mRNA to cDNA according to the manufacturer’s instructions. Primer sequences used in this study was listed as followed: *IL-1β* forward: 5’-ATGATGGCTTATTACAGTGGCAA-3’, reverse: 5’-GTCGGAGATTCGTAGCTGGA-3’; *IL-18* forward: 5’-TCTTCATTGACCAAGGAAATCGG-3’, reverse: 5’-TCCGGGGTGCATTATCTCTAC-3’; *GSDMD* forward: 5’-GGACAGGCAAAGATCGCAG-3’, reverse: 5’-CACTCAGCGAGTACACATTCATT-3’; *GSDME* forward: 5’-TGCCTACGGTGTCATTGAGTT-3’, reverse: 5’-TCTGGCATGTCTATGAATGCAAA-3’; *NLRP3* forward: 5’-CGTGAGTCCCATTAAGATGGAGT-3’, reverse: 5’-CCCGACAGTGGATATAGAACAGA-3’; *ASC* forward: 5’-TGGATGCTCTGTACGGGAAG-3’, reverse: 5’-CCAGGCTGGTGTGAAACTGAA-3’; *Caspase1* forward: 5’-CCTTAATATGCAAGACTCTCAAGGA-3’, reverse: 5’-TAAGCTGGGTTGTCCTGCACT-3’; *UBC* forward: 5’-ATTTGGGTCGCGGTTCTTG-3’, reverse: 5’- TGCCTTGACATTCTCGATGGT − 3’;

### Western blotting

Immunoblotting was performed as described in Xu et al. study[35], treated cell were lysed in RIPA buffer and quantified using BCA protein assay Kit. 20ug of the total protein extract were subjected from SDS-PAGE and transferred onto PVDF. the membranes were blocked with PBST with 5 % milk for 1 h. after incubation overnight with indicated primary antibodies, further incubation with a secondary antibody conjugated horseradish peroxidase for 1 h at room temperature. After washing with three times for 5 min, the proteins were detected using an enhanced chemiluminescence (Thermo fisher, MA, USA) reagent and imaged with western blotting detection system (Bio-Rad, USA).

### LDH release assay

LDH was released from damaged cells into supernatant and evaluated for cell death or cytotoxicity, which was widely used to analyze LDH activities in supernatants of BGC823 and AGS cells treated in the experiment according to the manufacturer’s instructions according to LDH cytotoxicity assays (ab65393). Relative cell cytotoxicity was determined as (test sample–baseline cell death)/(high control–baseline cell death)×100 %. Maximum cell death was indicated by a positive control for 100 % cell death generated by lysis buffer from the kit, while baseline cell death was indicated by a negative control of 0 % cell death as a result of no treatment.

### Elisa assay

Gastric cancer cell of BGC823 and AGS were treated with or without famotidine for 48 h, and the supernatant were collected and centrifuged to remove cell debris. Cytokines of IL-1β and IL‐18 level in supernatant were measured and quantitated in indicated group by sandwich ELISA according to the manufacturer’s instructions.

### Statistical analysis

Data analysis was performed using GraphPad Prism V software (La Jolla, CA, USA). A p less than 0.05 was considered statistical difference. Statistical differences among groups were determined by Student’s t-test, one-way ANOVA was used to determine the significance for mRNA and intensity quantified.

## Results

### Famotidine induced cell pyroptosis in gastric cancer cells

**Fig. 1:**
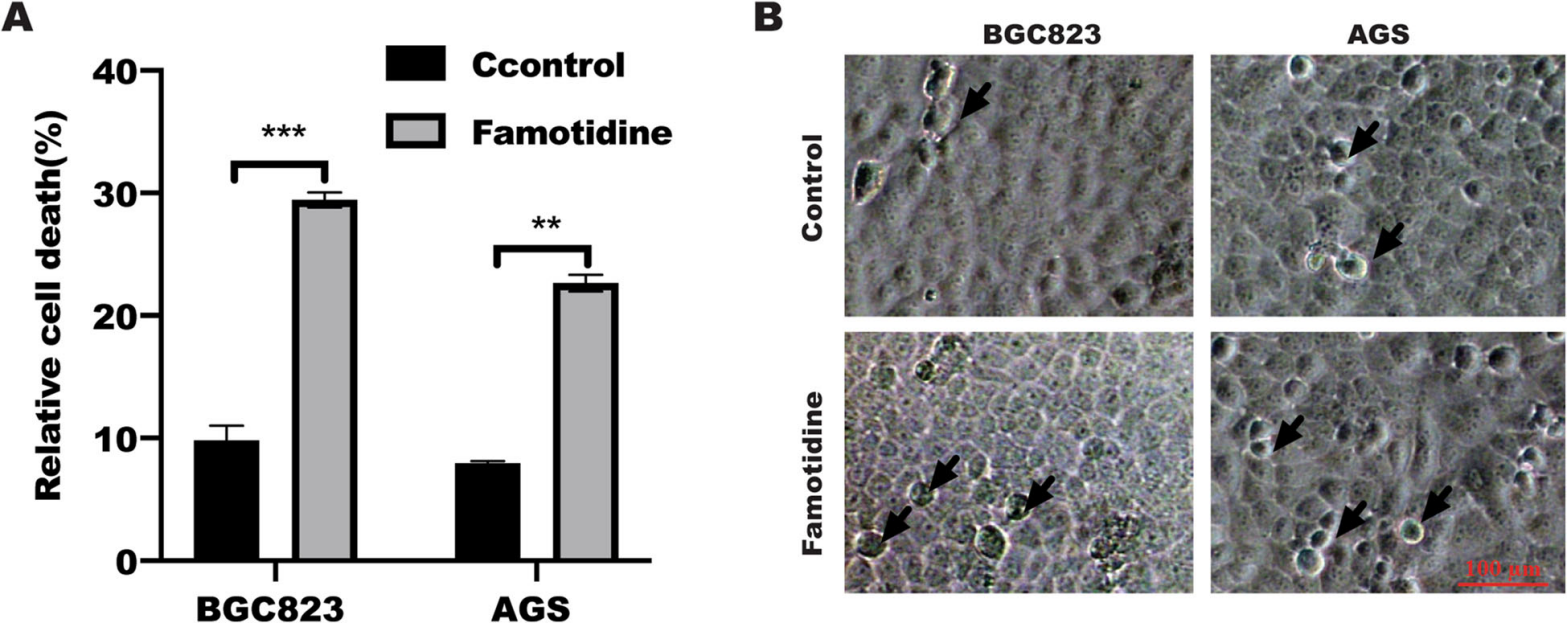
Famotidine induced cell pyroptosis. **A** Cytotoxicity of gastric cancer cells was measured by LDH assay in the culture supernatants from BGC823 and AGS cells treated with or without famotidine(300 μm) for 72 h. Data represented the mean ± s.d. of three independent experiments and were analyzed by the t test for significance versus the control group. ***P < 0.001, **P < 0.01 compared with control. **B** Representative microscopic images of BGC823 and AGS cells treated with or without 300 μm famotidine for 72 h. Black arrows indicate the characteristic balloon in the cell membrane. Scale bar is 100 μm

pyroptosis is a novel form of inflammatory programmed cell death and aggravate inflammatory reaction. To identify the potential function of famotidine in cell pyroptosis, LDH release assay were employed to detect in BGC823 and AGS cells treated with or without famotidine. As shown in Fig. 1 A, LDH release assay revealed cell death was significantly increased in BGC823 and AGS cells in response to famotidine stimulation in a time-dependent manner. Further analysis showed famotidine stimulation drastically increased the cell viability of BGC823 and AGS cells. in line with this, orphologically, a large number of dead cells were observed in BGC823 cells in absence with famotidine stimulation compared with that untreated control group, respectively(Fig. 1B).

### Famotidine activated GSDME and IL-18 release

**Fig. 2:**
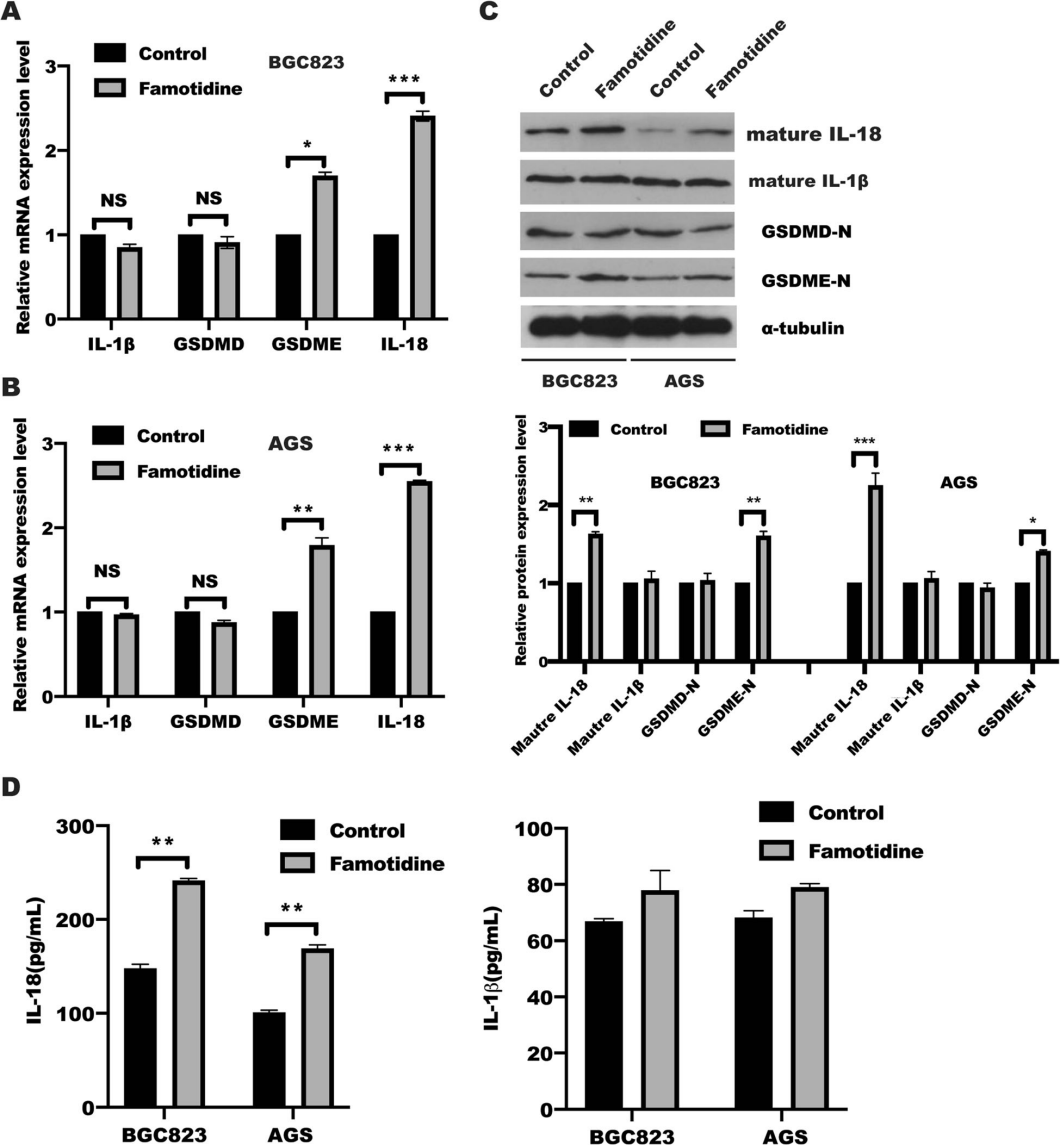
Famotidine contributed to GSDME activation and IL-18 release.** A** BGC-823 and **(B) **AGS cells were treated with or without famotidine for 72 h, mRNA levels of indicated genes were analyzed by RT-qPCR. Data represented the mean ± s.d. of three independent experiments and were analyzed by t test for significance versus Control group (0 μm), ***P < 0.001, **P < 0.01, *P < 0.05 versus control group(0 μm). NS: no significance. **C** BGC-823 and AGS cells were treated as indicated and at 72 h post-treatment, cells were harvested to extract total proteins. The SDS-PAGE and immunoblotting were performed to detect indicated proteins, and relative protein expression were quantified and analyzed by t test. Data represented the mean ± s.d. of three independent experiments and were analyzed by t test for significance versus Control group (0 μm), ***P < 0.001, **P < 0.01, *P < 0.05 versus control group(0 μm), the gels/blots and quantitative were processed in parallel. **D** BGC-823 and AGS cells were treated as described in C, the supernatant was collected. The level of IL-18 (left panel) and IL-1β (right panel) were measured by Elisa according to the instruction, respectively. Data represent the mean ± s.d. of three independent experiments and were analyzed by t test for significance versus Control group (0 μm), **P < 0.01 versus control group(0 μm)

The above results suggested that famotidine stimulation triggered cell pyroptosis in gastric epithelial cells, where gasdermin may serve as a pivotal executioner[36, 37], which attracted us to identify the pyroptosis-related genes responsible for famotidine treatment. our results demonstrated that famotidine treatment failed to induce GSDMD change, while the mRNA and protein level of GSDME expression was increased in BGC823 and AGS cells in response to famotidine treatment (Fig. 2 A-B), which further to release mature IL-18 secretion, but not IL-1β, determined by Elisa and western blotting (Fig. 2 C-D). these findings implied that famotidine promoted IL-18-mediated inflammation through enhanced GSDME-executed pyroptosis in gastric cancer cells.

### Famotidine regulated pyroptosis by NLRP3 inflammasome activation

**Fig. 3:**
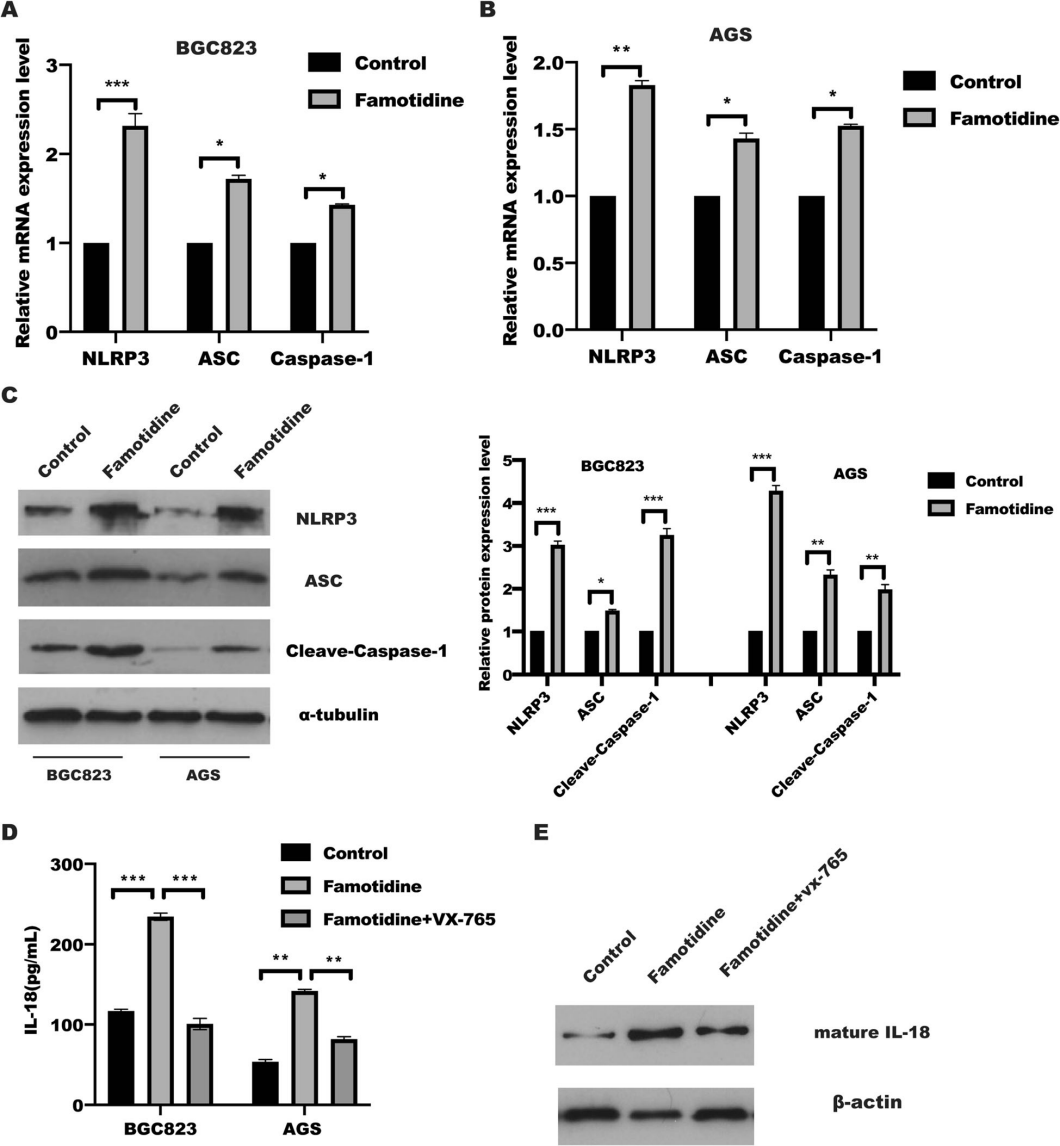
Famotidine triggered NLRP3 inflammasomes form.** A** BGC823 and **(B) **AGS cells were treated with or without famotidine for 72 h, mRNA levels of indicated inflammasome-related genes were analyzed by RT-qPCR. Data represented the mean ± s.d. of three independent experiments and were analyzed by t test for significance versus Control group (0 μm), ***P < 0.001, **P < 0.01, *P < 0.05 versus control group(0 μm). **C** BGC823 and AGS cells were treated as indicated and at 72 h post-treatment, cells were harvested to extract total proteins. The SDS-PAGE and immunoblotting were performed to detect indicated proteins, and relative protein expression were quantified and analyzed by t test. Data represented the mean ± s.d. of three independent experiments and were analyzed by t test for significance versus Control group (0 μm), ***P < 0.001, **P < 0.01, *P < 0.05 versus control group(0 μm). the gels/blots and quantitative were processed in parallel. **D** BGC823 and AGS cells were left untreated or treated with Caspase-1 inhibitor VX-765 for 1 h, followed by famotidine stimulation for another 71 h, the content of IL-18 level in indicated group were measured by IL-18 Elisa kit assay. Data represented the mean ± s.d. of three independent experiments and were analyzed by two-way ANOVA with multiple comparisons, followed by Bonferroni post hoc test for significance. ***P < 0.001, **P < 0.01. **E** the total protein was collected from above group in D, whole cell lysates were separated by SDS-PAGE and assayed with the antibodies against the indicated proteins. β-actin was determined to ensure equal loading. The quantified result was analyzed by two-way ANOVA with multiple comparisons, followed by Bonferroni post hoc test for significance. ***P < 0.001, **P < 0.01

Due to the NLRP3 inflammasome is currently the well-known characterized inflammasome and consists of NLRP3, apoptosis-associated speck-like protein containing a CARD (ASC), and caspase-1, which focused our attenuation to explore the effect of famotidine in inflammasome formation. Interestingly, real-time PCR assay demonstrated that NLRP3, ASC, and Caspase 1 expression were drastically increased in BGC823 and AGS cells in response to famotidine stimulation for 72 h (Fig. 3 A-B). In line with this, WB results also showed that famotidine treatment resulted in a significant upregulation of NLRP3, ASC, and Caspase-1 at protein expression level (Fig. 3 C), leading to NLRP3 inflammasome activation via association with the adaptor protein ASC, which in turn activates caspase-1 to cleave pro-IL-18 into active forms. What’s more, the caspase-1 inhibitor VX-765 treatment reversed the promotion effect of famotidine in IL-18 expression measured by Elisa and western blotting (Fig. 3D-E). However, these findings suggested that famotidine promoted cell pyroptosis to aggravate inflammation in gastric epithelial cells.

### Famotidine contributes cell pyroptosis by triggering ERK1/2

**Fig. 4:**
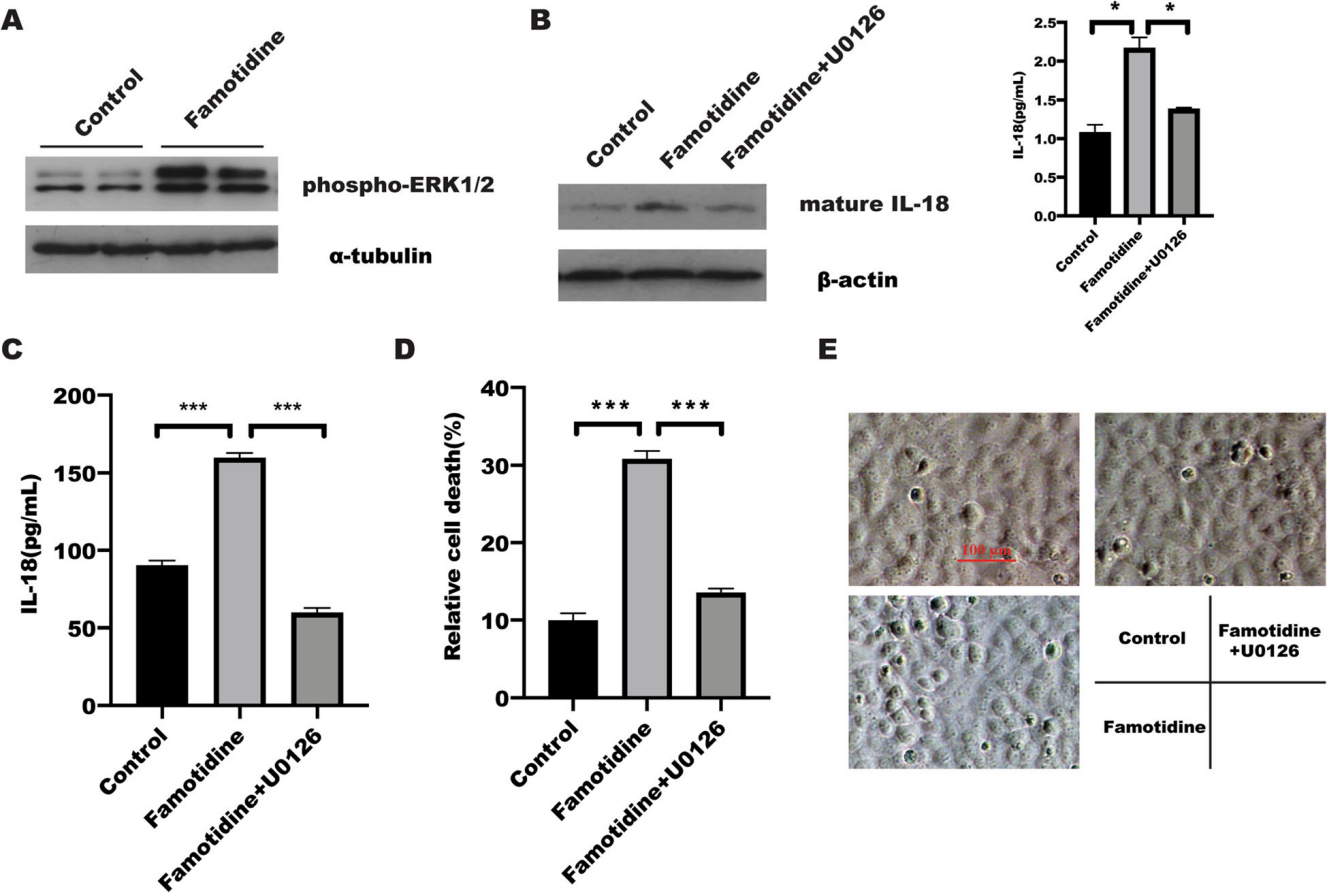
Famotidine treatment increased phosphorylation of ERK1/2.** A** after serum-starvation for 24 h, BGC823 and AGS cells were treated with or without famotidine for 1 h, respectively, phosphorylation of ERK1/2 and ERK1/2 level were detected by SDS-PAGE and immunoblotting. **B** BGC823 cells were treated with U0126 for 1 h, and followed by famotidine stimulation for 71 h, the mature IL-18 was detected in indicated group by western blotting, α-tubulin was taken as internal control. The level of IL-18 in supernatant was measured by Elisa assay. Data represented the mean ± s.d. of three independent experiments and were analyzed by One-ANOVA test for significance. *P < 0.05. **C** The LDH release was determined by LDH assay **(D)**, Data represented the mean ± s.d. of three independent experiments and were analyzed by One-ANOVA test for significance. ***P < 0.001. **E** Representative microscopic images of BGC823 cells treated as indicated for 72 h. Scale bar is 100 μm

The above results have suggested that famotidine induced cell pyroptosis in gastric cancer cells. However, the mechanism underlying famotidine-mediated pyroptosis remained to be identified. H_2_R ligands have proved to increase ERK phosphorylation through a dynamin or Gβγ pathway[38]. In line with these studies[39–42], our results showed that phosphorylation of ERK1/2 was significantly upregulated in AGS and BGC823 gastric cancer cells in response to famotidine stimulation (Fig. 4 A), while inhibition of ERK1/2 by U0126 was able to attenuated the promotion effect of famotidine on NLRP3 inflammasomes, leading to decrease IL-18 expression and LDH release determined by Elisa and western blotting(Fig. 4B-D). Phase contrast microscope images also showed that U0126 treatment led to a significant reversed effect of famotidine on cell death (Fig. 4E). taken together, these findings showed that famotidine promoted cell pyroptosis in BGC823 and AGS gastric cancer cells in dependent of EKR1/2.

## Discussion

Famotidine, clinically widespread used ligand acting at H_2_R[40], is sufficient to inhibit gastric acid secretion. However, the potential function of famotidine is gradually to be elucidate in various of disease. Previous study has revealed that another proton pump inhibitor omeprazole regulated lipid content in BGC823 cells, leading to reduce lipid content. In this study, we further showed famotidine treatment induced GSDME-, not GSDMD-, mediated cell pyroptosis by activation of NLRP3 inflammasome form, leading to increase Caspase-1 activation and IL-18 release in BGC823 and AGS cells. Further analysis showed phosphorylation of ERK1/2 was drastically enhanced in BGC823 and AGS cells in response to famotidine treatment, while inhibition of EKR1/2 activity led to a reversed effect of famotidine on IL-18. Despite a positive control that famotidine treatment in a cell line that does not express inflammasome is lacking to strong support our present findings, these results suggested the novel function of famotidine and implied a novel insight that a comprehensive consideration required for patient’s treatment.

Recently, the study has showed that famotidine treatment resulted in significant downregulation of LDH, TGF-β, bFGF and IL-9, respectively, and significant upregulation of Caspase-3 expression in diffuse large B-cell lymphoma[43]. Further analysis showed that famotidine prevented increment in oxidative stress markers and reduction of antioxidants during ischemia-reperfusion injury[44], in addition, the protective effect of famotidine was observed in neuroprotective[30]. However, no available direct reports about the famotidine in cell pyroptosis. Due to the study have showed saturated fatty acids have been reported to activate the NLRP3 inflammasome, resulting in IL-1β, secretion and aggravating inflammation[45]. We speculated that famotidine could promoted SREBP-1c mature, a transcription factor of fatty acids synthesis, and SREBP-1c could directly regulate NLRP3 expression. this famotidine-mediated fatty acids synthesis in NLRP inflammasome activation could be further addressed in our next work.

As shown in our study, famotidine treatment led to an upregulation of IL-18 release, a production of cell pyroptosis. NLRP3-mediated pyroptotic cell death and activation of Caspase-1 and GSDMD were the key event during cell pyroptosis[46]. As presented in this study, both NLRP3 and ASC expression were significantly increased in response to famotidine stimulation in BGC823 and AGS cells, leading to caspase-1 activation and IL-18 release, most important, famotidine failed to alter GSDMD expression, but induced GSDME expression, which is line with the report showed that GSDME rather than GSDMD is cleaved in chemotherapy drug-treated gastric cancer cells[15], suggesting the critical role of GSDME in famotidine-induced cell pyroptosis. However, the further work is required to elucidate whether other NLRPs inflammasomes and nonclassical caspase-4/-5-mediated pyroptosis involved in famotidine regulated pyroptosis. In addition to the function of famotidine on cell pyrotposis, the further investigation is also needed to explore the possible changes of apoptosis, necroptosis even ferroptosis, as well as the underlying mechanism.

It is well known that ERK1/2, a member of MAPK, pathway plays a crucial role in the regulation of cell proliferation[47, 48]. In the present study, stimulation with famotidine triggered the ERK 1/2 phosphorylation, while inhibition ERK1/2 activity could reverse the effect of famotidine on LDH and IL-18 expression, indicating that the famotidine regulated cell pyroptosis in ERK1/2-dependent manner. However, the following issue remained to be identified in our future work, including [1] The possible relationship between H2 recepotor, the target of famotidine, and ERK1/2 is remained to be identified. [2] to address the precise mechanism through which famotidine regulated phosphorylation of ERK1/2. [3] to reveal the downstream critical transcription factor of ERK1/2 involved in NLRP3 expression. [4] *in vivo* animal experiment and clinical sample analysis should be taken into the work. In addition to cell pyroptosis, the potential role of famotidine could be further addressed in gastric cancer in the future, such as tumor invasion, metabolism and angiogenesis.

## Conclusions

In summary, these findings extended the novelty function of famotidine and revealed a critical role of famotidine in gastric cancer cell pyroptosis to aggravate inflammation, which may be not suitable for gastric cancer treatment, implying a more comprehensive consideration is needed in management of gastric cancer.

## Data Availability

The datasets generated during and/or analyses during the current study are available from the corresponding author on reasonable request.

## Abbreviations

ASC: apoptosis-associated speck-like protein containing a CARD
DNL: *De novo* lipogenesis
ERK1/2: Extracellular signal-regulated kinase
FASN: fatty acid synthase
GC: gastric cancer
GSH: glutathione
GECs: Gastric epithelial cells
GSDM: gasdermin
H. pylori: Helicobacter pylori
IL: Interleukin
IHC: Immunohistochemistry
LDH: lactate dehydrogenase
MALT: mucosa-associated lymphoid tissue
NLRP3: NOD-like receptor family, pyrin domain containing 3
PPI: Proton Pump Inhibitor
QPCR: quantitative polymerase chain reaction
WB: western blotting

## References

[1] Lordick F, Lorenzen S, Yamada Y, Ilson D (2014). Optimal chemotherapy for advanced gastric cancer: is there a global consensus?. Gastric Cancer.

[2] Hu Y, Ma Z, He Y, Liu W, Su Y, Tang Z (2017). LncRNA-SNHG1 contributes to gastric cancer cell proliferation by regulating DNMT1. Biochem Biophys Res Commun.

[3] Akino K, Toyota M, Suzuki H, Imai T, Maruyama R, Kusano M (2007). Identification of DFNA5 as a target of epigenetic inactivation in gastric cancer. Cancer Sci.

[4] Torre LA, Bray F, Siegel RL, Ferlay J, Lortet-Tieulent J, Jemal A (2015). Global cancer statistics, 2012. CA Cancer J Clin.

[5] Chen W, Zheng R, Baade PD, Zhang S, Zeng H, Bray F (2016). Cancer statistics in China, 2015. CA Cancer J Clin.

[6] Wu WK, Cho CH, Lee CW, Fan D, Wu K, Yu J (2010). Dysregulation of cellular signaling in gastric cancer. Cancer Lett.

[7] Japanese Gastric Cancer A. Japanese gastric cancer treatment guidelines 2018 (5th edition). Gastric Cancer. 2021;24(1):1–21. 10.1007/s10120-020-01042-y PMC7790804 32060757

[8] Chen N, Han X, Yin B, Bai X, Wang Y (2021). FGD5 facilitates tumor growth by regulating EGFR ubiquitination in gastric cancer. Biochem Biophys Res Commun.

[9] Yang H, Liu G, Zhao H, Dong X, Yang Z. Inhibiting the JNK/ERK signaling pathway with geraniol for attenuating the proliferation of human gastric adenocarcinoma AGS cells. J Biochem Mol Toxicol. 2021:e22818. 10.1002/jbt.22818 34075659

[10] Zhou CB, Fang JY (2019). The role of pyroptosis in gastrointestinal cancer and immune responses to intestinal microbial infection. Biochim Biophys Acta Rev Cancer.

[11] Ren N, Jiang T, Wang C, Xie S, Xing Y, Piao D (2020). LncRNA ADAMTS9-AS2 inhibits gastric cancer (GC) development and sensitizes chemoresistant GC cells to cisplatin by regulating miR-223-3p/NLRP3 axis. Aging (Albany NY).

[12] Deng BB, Jiao BP, Liu YJ, Li YR, Wang GJ (2020). BIX-01294 enhanced chemotherapy effect in gastric cancer by inducing GSDME-mediated pyroptosis. Cell Biol Int.

[13] Wang WJ, Chen D, Jiang MZ, Xu B, Li XW, Chu Y (2018). Downregulation of gasdermin D promotes gastric cancer proliferation by regulating cell cycle-related proteins. J Dig Dis.

[14] Li G, Zhu L, Cao Z, Wang J, Zhou F, Wang X (2018). A New Participant in the Pathogenesis of Alcoholic Gastritis: Pyroptosis. Cell Physiol Biochem.

[15] Wang Y, Yin B, Li D, Wang G, Han X, Sun X (2018). GSDME mediates caspase-3-dependent pyroptosis in gastric cancer. Biochem Biophys Res Commun.

[16] Fuchs Y, Steller H (2015). Live to die another way: modes of programmed cell death and the signals emanating from dying cells. Nat Rev Mol Cell Biol.

[17] Tang L, Lu C, Zheng G, Burgering BM (2020). Emerging insights on the role of gasdermins in infection and inflammatory diseases. Clin Transl Immunology.

[18] Shi J, Gao W, Shao F, Pyroptosis (2017). Gasdermin-Mediated Programmed Necrotic Cell Death. Trends Biochem Sci.

[19] He Y, Amer AO (2014). Microbial modulation of host apoptosis and pyroptosis. Front Cell Infect Microbiol..

[20] Jesenberger V, Procyk KJ, Yuan J, Reipert S, Baccarini M (2000). Salmonella-induced caspase-2 activation in macrophages: a novel mechanism in pathogen-mediated apoptosis. J Exp Med.

[21] Kelk P, Johansson A, Claesson R, Hanstrom L, Kalfas S (2003). Caspase 1 involvement in human monocyte lysis induced by Actinobacillus actinomycetemcomitans leukotoxin. Infect Immun.

[22] Cervantes J, Nagata T, Uchijima M, Shibata K, Koide Y (2008). Intracytosolic Listeria monocytogenes induces cell death through caspase-1 activation in murine macrophages. Cell Microbiol.

[23] Shi J, Zhao Y, Wang K, Shi X, Wang Y, Huang H (2015). Cleavage of GSDMD by inflammatory caspases determines pyroptotic cell death. Nature.

[24] Galluzzi L, Vitale I, Aaronson SA, Abrams JM, Adam D, Agostinis P (2018). Molecular mechanisms of cell death: recommendations of the Nomenclature Committee on Cell Death 2018. Cell Death Differ.

[25] Newton K, Wickliffe KE, Dugger DL, Maltzman A, Roose-Girma M, Dohse M (2019). Cleavage of RIPK1 by caspase-8 is crucial for limiting apoptosis and necroptosis. Nature.

[26] O’Donnell MA, Perez-Jimenez E, Oberst A, Ng A, Massoumi R, Xavier R (2011). Caspase 8 inhibits programmed necrosis by processing CYLD. Nat Cell Biol.

[27] Li S, Liang X, Ma L, Shen L, Li T, Zheng L (2018). MiR-22 sustains NLRP3 expression and attenuates H. pylori-induced gastric carcinogenesis. Oncogene.

[28] Chen P, Li L, Wang H, Zhao J, Cheng Y, Xie J (2020). Omeprazole, an inhibitor of proton pump, suppresses De novo lipogenesis in gastric epithelial cells. Biomed Pharmacother.

[29] Zhou Y, Chen S, Yang F, Zhang Y, Xiong L, Zhao J (2021). Rabeprazole suppresses cell proliferation in gastric epithelial cells by targeting STAT3-mediated glycolysis. Biochem Pharmacol.

[30] Unal G, Dokumaci AH, Ozkartal CS, Yerer MB, Aricioglu F (2019). Famotidine has a neuroprotective effect on MK-801 induced toxicity via the Akt/GSK-3beta/beta-catenin signaling pathway in the SH-SY5Y cell line. Chem Biol Interact.

[31] Potnuri AG, Allakonda L, Saheera S. Involvement of Histamine 2 Receptor in Alpha 1 Adrenoceptor Mediated Cardiac Hypertrophy and Oxidative Stress in H9c2 Cardio Myoblasts. J Cardiovasc Transl Res. 2020. 10.1007/s12265-020-09967-6 32385805

[32] Yokoyama M, Yokoyama A, Mori S, Takahashi HK, Yoshino T, Watanabe T (2004). Inducible histamine protects mice from P. acnes-primed and LPS-induced hepatitis through H2-receptor stimulation. Gastroenterology.

[33] Li C, Qiu J, Xue Y (2021). Low-dose Diosbulbin-B (DB) activates tumor-intrinsic PD-L1/NLRP3 signaling pathway mediated pyroptotic cell death to increase cisplatin-sensitivity in gastric cancer (GC). Cell Biosci.

[34] Zhang S, Xu W, Wang H, Cao M, Li M, Zhao J (2019). Inhibition of CREB-mediated ZO-1 and activation of NF-kappaB-induced IL-6 by colonic epithelial MCT4 destroys intestinal barrier function. Cell Prolif.

[35] Xu W, Zhang Z, Zou K, Cheng Y, Yang M, Chen H (2017). MiR-1 suppresses tumor cell proliferation in colorectal cancer by inhibition of Smad3-mediated tumor glycolysis. Cell Death Dis.

[36] Poh L, Kang SW, Baik SH, Ng GYQ, She DT, Balaganapathy P (2019). Evidence that NLRC4 inflammasome mediates apoptotic and pyroptotic microglial death following ischemic stroke. Brain Behav Immun.

[37] Zhang D, Qian J, Zhang P, Li H, Shen H, Li X (2019). Gasdermin D serves as a key executioner of pyroptosis in experimental cerebral ischemia and reperfusion model both in vivo and in vitro. J Neurosci Res.

[38] Werry TD, Sexton PM, Christopoulos A. “Ins and outs” of seven-transmembrane receptor signalling to ERK. Trends Endocrinol Metab. 2005;16(1):26–33. 10.1016/j.tem.2004.11.008 15620546

[39] Kim D, Cho S, Castano MA, Panettieri RA, Woo JA, Liggett SB (2019). Biased TAS2R Bronchodilators Inhibit Airway Smooth Muscle Growth by Downregulating Phosphorylated Extracellular Signal-regulated Kinase 1/2. Am J Respir Cell Mol Biol.

[40] Alonso N, Zappia CD, Cabrera M, Davio CA, Shayo C, Monczor F (2015). Physiological implications of biased signaling at histamine H2 receptors. Front Pharmacol.

[41] Luo T, Chen B, Zhao Z, He N, Zeng Z, Wu B (2013). Histamine H2 receptor activation exacerbates myocardial ischemia/reperfusion injury by disturbing mitochondrial and endothelial function. Basic Res Cardiol.

[42] Nonaka T, Mio M, Doi M, Tasaka K (1992). Histamine-induced differentiation of HL-60 cells. The role of cAMP and protein kinase A. Biochem Pharmacol.

[43] Hegazy SK, El-Haggar SM, Alhassanin SA, El-Berri EI (2021). Comparative randomized trial evaluating the effect of proton pump inhibitor versus histamine 2 receptor antagonist as an adjuvant therapy in diffuse large B-cell lymphoma. Med Oncol.

[44] Tanriverdi HI, Senel U, Gevrek F, Akbas A. Protective effect of famotidine on ischemia-reperfusion injury following testicular torsion in rats. J Pediatr Urol. 2020. 10.1016/j.jpurol.2020.09.019 33046373

[45] Robblee MM, Kim CC, Porter Abate J, Valdearcos M, Sandlund KL, Shenoy MK (2016). Saturated Fatty Acids Engage an IRE1alpha-Dependent Pathway to Activate the NLRP3 Inflammasome in Myeloid Cells. Cell Rep.

[46] Zhou L, Liu T, Huang B, Luo M, Chen Z, Zhao Z, et al. Excessive deubiquitination of NLRP3-R779C variant contributes to very-early-onset inflammatory bowel disease development. J Allergy Clin Immunol. 2020. 10.1016/j.jaci.2020.09.003 32941940

[47] Kim EK, Choi EJ (2010). Pathological roles of MAPK signaling pathways in human diseases. Biochim Biophys Acta.

[48] Ballif BA, Blenis J (2001). Molecular mechanisms mediating mammalian mitogen-activated protein kinase (MAPK) kinase (MEK)-MAPK cell survival signals. Cell Growth Differ.

